# Rare Variants in Ischemic Stroke: An Exome Pilot Study

**DOI:** 10.1371/journal.pone.0035591

**Published:** 2012-04-20

**Authors:** John W. Cole, O. Colin Stine, Xinyue Liu, Abhishek Pratap, Yuching Cheng, Luke J. Tallon, Lisa K. Sadzewicz, Nicole Dueker, Marcella A. Wozniak, Barney J. Stern, James F. Meschia, Braxton D. Mitchell, Steven J. Kittner, Jeffrey R. O'Connell

**Affiliations:** 1 Veterans Administration Medical Center, Baltimore, Maryland, United States of America; 2 University of Maryland School of Medicine, Baltimore, Maryland, United States of America; 3 Mayo Clinic, Jacksonville, Florida, United States of America; The University of Hong Kong, Hong Kong

## Abstract

The genetic architecture of ischemic stroke is complex and is likely to include rare or low frequency variants with high penetrance and large effect sizes. Such variants are likely to provide important insights into disease pathogenesis compared to common variants with small effect sizes. Because a significant portion of human functional variation may derive from the protein-coding portion of genes we undertook a pilot study to identify variation across the human exome (i.e., the coding exons across the entire human genome) in 10 ischemic stroke cases. Our efforts focused on evaluating the feasibility and identifying the difficulties in this type of research as it applies to ischemic stroke. The cases included 8 African-Americans and 2 Caucasians selected on the basis of similar stroke subtypes and by implementing a case selection algorithm that emphasized the genetic contribution of stroke risk. Following construction of paired-end sequencing libraries, all predicted human exons in each sample were captured and sequenced. Sequencing generated an average of 25.5 million read pairs (75 bp×2) and 3.8 Gbp per sample. After passing quality filters, screening the exomes against dbSNP demonstrated an average of 2839 novel SNPs among African-Americans and 1105 among Caucasians. In an aggregate analysis, 48 genes were identified to have at least one rare variant across all stroke cases. One gene, *CSN3*, identified by screening our prior GWAS results in conjunction with our exome results, was found to contain an interesting coding polymorphism as well as containing excess rare variation as compared with the other genes evaluated. In conclusion, while rare coding variants may predispose to the risk of ischemic stroke, this fact has yet to be definitively proven. Our study demonstrates the complexities of such research and highlights that while exome data can be obtained, the optimal analytical methods have yet to be determined.

## Introduction

Stroke is the fourth leading cause of death and the leading cause of disability in the US [1], requiring expenditures near $70 billion annually [2]. Stroke prevention and treatment efforts are hampered by a lack of understanding of the basic etiologic mechanisms. Even in clinically well-defined stroke subtypes such as lacunar stroke, the genetic architecture remains uncertain and could be determined by common variants with small effect sizes, rare variants with large effect sizes, or a combination of common and rare variants. The genome-wide association (GWA) approach is designed to detect common disease-associated variants, but is not designed to detect rare variants that occur infrequently in the population or private to the affected individual. The rare variant hypothesis posits that multiple rare variants, within the same gene or pathway, may lead to disease. Identification of rare variants may lead to critically important insights about disease etiology through implication of new genes and/or pathways. At this time full-genome sequencing remains cost prohibitive; however, the genetic variation in the protein-coding portion of genes can be cost effectively studied and is of significant interest in the study of human health. The ‘exome’, or coding exons, are thought to harbor a significant portion of human functional variation and contain many rare variants of high penetrance. The human exome refers to the 1% of the human genome that codes for proteins and consists of approximately 50 Mb covering ∼25,000 genes and includes both exons and microRNAs [3]. Our focus on the exome is based upon the belief that the exome harbors a significant portion of human functional variation. Variation within the exome is responsible for most Mendelian diseases, primarily mutations that cause amino acid substitutions, including changes to nonsense codons (∼60%) [4], [5], but also small indels (insertions/deletions) in genes (∼25%).

### Rare variants and disease

Several studies have successfully employed exome sequencing to identify rare mutations that are responsible for Mendelian disease. In a proof-of-principle study, four affected individuals with Freeman-Sheldon syndrome (FSS - OMIM: #193700 - accessed 2010 July) and eight HapMap controls underwent exome were sequencing to demonstrate that that this technique could identify the known mutations in the *MYH3* gene that cause the disease [6]. In another study, exome sequencing identified the previously unknown causative gene *DHODH* for Miller Syndrome (OMIM: #263750 - accessed 2010 July), a rare autosomal recessive Mendelian disease characterized by an under-sized jaw, droopy eyes, cleft lip or palate, and incomplete or unusual limb development [7].

While these examples demonstrate the usefulness of the exome approach in identifying rare variants associated with Mendelian disease, other studies are now revealing the role of rare variants in complex disease. In one such study, rare variants were determined to be associated with development of the well-established complex disease of hypertension [8]. In this study, investigators evaluated for the presence of rare loss-of-function mutations in genes coding for transporter proteins involved in renal sodium handling. Deficient homozygotes develop rare Mendelian disorders known as Gitelman's (OMIM: **#263800** - accessed 2010 July) and Bartter's (OMIM: **#607364** - accessed 2010 July) syndromes, both of which are characterized by low blood pressures. The investigators demonstrated that heterozygote mutation carriers had significantly lower mean long-term values for systolic (−6.3 mm Hg; p = 0.0009) and diastolic (−3.4 mm Hg; p = 0.003) BP and a 59% (95% confidence interval: 23% to 71%; p<0.003) lower risk of developing hypertension by age 60 years. In summary, individuals in the lower blood pressure ‘tail’ of the overall blood pressure distribution were found to have rare coding genetic variants influencing renal salt handling that contributed to blood pressure variation at the population level.

### Young-onset stroke is well suited for studies of the exome to identify rare variants

Various studies of complex diseases indicate that young onset disease is more likely due to multiple rare exonic variants with large effects as compared to late-onset disease. As an example, studies identified rare mutations in the *parkin* gene in the PARK2 locus that cause sporadic early-onset parkinsonism [9]. Subsequently, other studies identified other *parkin* variants that play a role in the common late-onset forms of Parkinson disease (age of onset >45 years) [10]. Hence, identification of rare mutations in genes that cause early-onset disease can identify genes associated with the more common, later-onset form of the disease.

Regarding stroke, twin studies and familial aggregation studies suggest a stronger genetic contribution to early onset than later onset stroke. Reports from the National Academy of Sciences-National Research Council Twin Registry showed a greater concordance in monozygotic twins relative to dizygotic twins when the twin cohort was younger [11], [12] Evidence from prior research, including meta-analyses [13], has consistently supported a greater degree of familial aggregation and a greater inherited component of stroke among early-onset compared to late-onset ischemic stroke cases. These findings were replicated using our own young stroke dataset, which demonstrated that siblings of stroke cases had more than four times the risk of stroke compared to siblings of controls (OR, 4.17; 95% CI, 1.9 to 8.8) and mothers of stroke cases had twice the risk of stroke compared to mothers of controls (OR, 2.02; 95% CI, 1.4 to 3.0) [14]. Furthermore, the association between stroke in probands and family history of stroke was strongest among women ages 15–24 years (OR, 2.5; 95% CI, 0.4 to 15.1), and diminished with increasing proband age, (OR, 1.6; 95% CI, 0.8 to 3.3 among women 25–34 yrs and OR, 1.5; 95% CI, 1.1 to 1.9 among women 35–49 yrs). Taken in aggregate, the evidence supports the point of view that the extreme disease phenotype, young age-of-onset stroke, has more familial aggregation and is likely more genetically driven than later onset forms of the disease.

In summary, this background information demonstrates the usefulness of the exome approach in identifying rare variants associated with Mendelian disease and also implies that by studying individuals in the extreme phenotypic tails in common complex disease, one can identify rare variants that have population-level effects on disease risk. While young-onset stroke is clearly not a Mendelian transmitted disease, rare variants of high penetrance could conceivably contribute to risk among the young-onset stroke phenotype. As such, a young age-of-onset can be considered as an extreme tail of the liability threshold distribution which is also often a characteristic of Mendelian disease. In this report, we describe our pilot study efforts to identify rare variants associated with ischemic stroke and the complexities of such research. We believe that sequencing will ultimately identify rare risk variants and that exome sequencing, given its ability to identify rare variants of high penetrance, is an excellent methodology to begin these efforts.

## Methods

### Ethics Statement

All human subjects research presented herein has been reviewed and approved by the Institutional Review Boards of the University of Maryland and the Mayo Clinic. Written informed consent was obtained from all participants involved in the study.

### Source study populations

Ten ischemic stroke subjects were selected for exome sequencing and analyses; 8 African-American participants from the Genetics of Early Onset Stroke Study and 2 European-Caucasian participants from the Siblings with Ischemic Stroke Study.

### Genetics of Early Onset Stroke (GEOS) Study

GEOS is a population-based study of ischemic stroke among Caucasian and African-American young adults in the Baltimore-Washington area that is composed of patients and controls recruited in 3 different time periods: Stroke Prevention in Young Women-1 (SPYW-1) conducted from 1992–1996, Stroke Prevention in Young Women-2 (SPYW-2) conducted from 2001–2003, and Stroke Prevention in Young Men (SPYM) conducted from 2003–2007. The study population (see **Table 1**) has slightly more men than women, and slightly more Caucasians than African-Americans.

**Table 1:** GEOS population characteristics by case-control status.

	Cases(n = 889)	Controls(n = 927)	*P*-value
Age (mean ± SD, years)	41.3±6.9	39.6±6.8	<0.001
Female (%)	41.5	43.6	0.37
Self-reported race (%)			0.22
European ancestry	52.42	56.42	
African ancestry	42.41	38.51	
Others	5.17	5.07	
TOAST Subtype (%)		---	---
Cardioembolic	20.0		
Large Artery	7.1		
Lacunar	16.1		
Other Known Causes	6.5		
Undetermined Causes	50.3		
Hypertension (%)	42.7	19.2	<0.001
Diabetes mellitus (%)	16.7	5.1	<0.001
Angina/MI (%)	5.3	0.7	<0.001
Current smokers (%)	42.5	28.6	<0.001

*Case participants* were hospitalized with a first cerebral infarction identified by discharge surveillance from one of 59 hospitals in the greater Baltimore-Washington area and direct referral from regional neurologists. SPYW-1 included cases ages 15–44 years recruited within one year of stroke and was designed with a 1∶2 case-to-control ratio. SPYW-2 and SPYM included cases ages 15–49 recruited within three years of stroke and was designed with a 1∶1 case-to-control ratio. The abstracted hospital records of cases were reviewed and adjudicated for ischemic stroke subtype by a pair of neurologists according to previously published procedures [15], with disagreements resolved by a third neurologist. The ischemic stroke subtype classification system retains information on all probable and possible causes, and is reducible to the more widely used TOAST [16] system that assigns each case to a single category. Monongenetic forms of stroke were excluded with further exclusions based upon the criteria used in the Siblings With Ischemic Stroke Study [17]. Summary results of the final TOAST classification for GEOS cases can be seen in **Table 1**.

*Control participants* without a history of stroke were identified by random-digit dialing. Controls were balanced to cases by age and region of residence in each study and were additionally balanced for ethnicity in SPYW-2 and SPYM.

For both *cases and controls* risk factors including hypertension, diabetes mellitus, and myocardial infarction were determined by asking the study participant (or his/her proxy if the participant was unable to answer) if he/she had ever been told by a physician that he/she had the condition. Similarly, age, race, ethnicity, oral contraceptive use, and smoking history were determined by subject or proxy report. Less than 5% of participants had proxy interviews or assisted interviews. Cigarette smoking information included: ever smoker, current smoker, former smoker, number of years smoking, average number of cigarettes smoked during smoking years, and pack-years of smoking. (See **Table 1**).

### Siblings with Ischemic Stroke Study (SWISS)

The aim of SWISS was to test the hypothesis that the human genome contains chromosomal regions associated with ischemic stroke by means of genome-wide scanning in DNA samples collected from 300 sibling pairs concordant for ischemic stroke and from 200 discordant siblings [17]. DNA collection began October of 2000 with the trial completed in 2010. Analyses are ongoing. The population study consists of three classes of participants including: probands, concordant siblings, and discordant siblings - each with their own inclusion and exclusion criteria. Probands were required to experience at least one ischemic stroke episode confirmed by either head CT or MRI completed within 7 days of symptom onset. In addition they were required to have at least one living full sibling with a history of ischemic stroke. There is no time limit on how long ago the stroke may have occurred in the sibling(s). Concordant siblings were required to have at least one radiographically-verified ischemic stroke episode, in addition to one living full sibling enrolled as a proband in SWISS. Discordant siblings were required to have no history of ischemic stroke, transient ischemic attack (TIA), or stroke symptoms, and must have at least two full siblings with a history of ischemic stroke who were enrolled in SWISS.

#### Exclusion Criteria

Patients whose strokes occurred within 48 hours of an invasive cerebrovascular or cardiovascular procedure, a catheter-based procedure on carotid or coronary arteries, carotid endarterectomy, heart valve surgery, or thoracic or thoracoabdominal aortic aneurysm repair, whose ischemic strokes occur within 60 days of nontraumatic subarachnoid hemorrhage (SAH), who have had brain-biopsy-proven central nervous system vasculitis at any time, who have Cerebral Autosomal Dominant Arteriopathy with Subcortical Infarcts and Leukoencephalopathy (CADASIL), Fabry disease, homocystinuria, mitochondrial encephalopathy with lactic acidosis and stroke-like episodes (MELAS), or sickle -cell anemia, or who had a mechanical aortic or mitral valve, or bacterial endocarditis at the time of stroke onset, were excluded from the Proband and Concordant Sibling groups. Patients who gave a positive answer to any of the 8 questions on the Questionnaire for Verifying Stroke-Free Status (QVSFS), or who deemed unreliable historians by the study interviewer due to moderate or severe impairment of speech, language, hearing, or memory, were be excluded from the Discordant Sibling group.

#### Patient Involvement

Probands: Prior to enrollment, patients' medical records and test results were reviewed by a study neurologist to confirm the diagnosis of ischemic stroke. Eligible patients were classified by stroke subtype, according to the TOAST criteria [16]. Probands then mailed invitations to participate in SWISS to their siblings. Discordant Siblings: Persons who responded positively to the SWISS invitation were given a Questionnaire for Verifying Stroke-Free Status (QVSFS) and a brief medical history was obtained via telephone interview. Patients who receive a QVSFS score >0 were considered for inclusion in the Concordant Siblings group. Concordant Siblings: Persons who respond positively to the SWISS invitation had their medical records requested and reviewed by investigators in order to verify the stroke diagnosis. Once an eligible proband had at least one verified concordant sibling, blood was collected by a home health care agency from all enrolled family members for genetic analysis.

### Exome Pilot Study Population

Considerable thought was given as to which ten cases to include in our analyses, as the goal of this pilot was to not only to demonstrate the effective use of exome methodologies, but that these exomes would then also be combined with additional future exomes for further analyses. Additionally, future collaborative efforts between the GEOS and SWISS studies were also considered. Having two SWISS European-Caucasian samples run on the University of Maryland platform and through the bioinformatics pipeline would allow for future quality control evaluations on SWISS samples undergoing exome sequencing elsewhere.

As described in the Introduction, prior successes to identify causative disease variants implementing exome analyses have centered on the use of a highly defined phenotype. Given the heterogeneity of stroke and numerous stroke subtypes, each of which might be considered as a separate complex disease, implementing this methodology in our stroke population was somewhat more difficult. In an effort to maximize the genetic contribution to stroke risk, we limited our case selection to only one gender (male), cases with an early age-of-onset, a positive family history of stroke, and the presence of no (or minimal) standard stroke risk factors. We also worked to limit the stroke subtypes selected, although in retrospect *selecting only one subtype* would have been the optimal methodology. As seen in **Table 2**, the 10 stroke cases included 8 African-Americans (from GEOS) and 2 European-Caucasians (SWISS samples); the TOAST subtypes included 5 lacunar, 2 cryptogenic, 2 dissection, and 1 cardioembolic stroke.

**Table 2:** Characteristics of the 10 male stroke cases implemented in the pilot study.

Study-ID	Ethnicity	Age	Positive Family History	Hypertension	Diabetes	Smoking	TOAST Stroke Type
M-006	African American	46	No	No	No	Former	Dissection
M-0114	African American	48	Yes	Yes	Yes	Never	Dissection
M-0379	African American	44	Yes	No	No	Never	Cryptogenic
M-0432	African American	42	Yes	No	No	Current	Lacunar
M-0823	African American	41	Yes	Yes	No	Never	Cardioembolic
M-1012	African American	45	Yes	No	No	Never	Lacunar
M-1096	African American	42	Yes	No	No	Never	Cryptogenic
M-1107	African American	47	Yes	Yes	No	Never	Lacunar
SW-393	Caucasian	57	Yes	Yes	No	Never	Lacunar
SW-708	Caucasian	61	Yes	Yes	No	Former	Lacunar

### Existing GWAS data

Here we briefly describe our recently completed GWAS on the GEOS study population, as some of these results were utilized to inform on our exome analyses. Our group recently implemented the Illumina 1 M Quad GWAS chip on the GEOS population (including the 8 GEOS exome samples). The SNPs included on this chip were chosen based upon allele frequencies greater than 5% within HapMap datasets; therefore, rare variants were specifically excluded. Following extensive data cleaning procedures, GWAS analyses were performed with a manuscript detailing these results recently published [18]. To summarize, we genotyped 1 million SNPs in our biracial GEOS population consisting of 889 ischemic stroke cases and 927 control subjects, aged 15–49 years, and imputed up to approximately 1.4 millions SNPs using HapMap3 reference panel to test their associations with stroke risk using logistic regression models adjusting for age, recruitment stages and population structure. We did not identify any associations that achieved stringent genome-wide significance. The most strongly associated SNPs were rs2304556 (P = 1.18×10-7) in the intron of *FMNL2* (HGNC:18267), which encodes a formin-related protein, and rs1986743 (P = 2.65×10-7) in the intron of *ARL6IP6* (HGNC:24048), which encodes ADP-ribosylation-like factor 6 interacting protein 6. As such, no SNP attained genome wide significance in our modestly-sized GWAS, supporting the notion that a few common variants do not account for the majority of stroke risk.

Having GWAS data on our exome cases allowed us to assess and compare genotype calls from a quality control standpoint (see **Table S1**). Also, as related to our future plans, GWAS data from these same study subjects can be utilized to select the optimal control subjects from other pre-existing publically available exome controls samples (e.g. NHLBI GO ESP available at dbGAP) to minimize confounding secondary to admixture.

The GWAS data used in these analyses is available at database of Genotypes and Phenotypes (dbGaP) (http://www.ncbi.nlm.nih.gov/projects/gap/cgi-bin/study.cgi?study_id=phs000292.v1.p1; dbGaP Study Accession: phs000292.v1.p1 - accessed 2012 January).

### Exome Sequencing Methodology

Sequencing library construction, exome capture, sequencing, and initial analyses for our study was performed by the Genomics Resource Center (GRC) within the Institute for Genome Sciences (IGS) at the University of Maryland School of Medicine. Construction of paired-end Illumina sequencing libraries were performed as prescribed by the manufacturer. These libraries will include unique 6 bp index sequences to enable multiplexed sequencing of more than one library per flowcell lane. Following library construction, Agilent's SureSelect technology was utilized to target capture of all predicted human exons in each sample for high-throughput sequencing using the Illumina Genome Analyzer IIx (GAIIx) platform. The method utilizes custom-designed, biotinylated RNA oligonucleotides for hybridization to the target sequence [19]. We implemented the Human All Exon kit using 120-mer baits designed to tile across more than 37 Mbp of human genomic sequence, including all CDS-annotated and GENCODE exons and more than one thousand additional non-coding RNAs [3].

Following hybridization of library fragments to the RNA baits, the bound fragments were captured by streptavidin-coated magnetic beads, and unbound fragments were washed away. The RNA was then digested, leaving captured library fragments ready for sequencing. The captured library fragments were sequenced using an Illumina Genome Analyzer IIx. We sequenced 100 bp from both ends of each library fragment. Each lane of Illumina GAIIx sequencing generated 80–100 million high-quality, passed-filter sequence read pairs. Of these, approximately 75% were uniquely mappable reads with an estimated 70–90% of the uniquely mappable reads falling on or within 200 bp of a targeted exon. This sequence coverage yielded at least 20× sequence coverage for more than 75% of targeted exons, leading to high confidence SNP calls with low false positive rates [3], [20].

**Figure 1:**
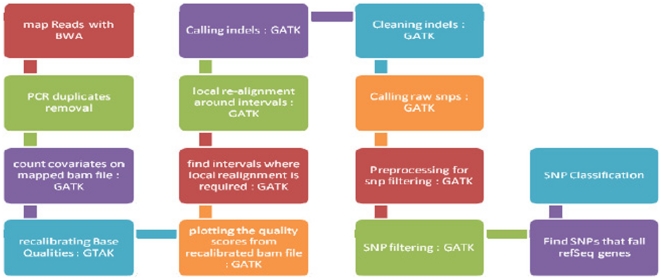
Summary of the pipeline steps.

In addition to performing the exome capture and sequencing, the Genomics Resource Center provided bioinformatics support for the project. Raw data from the sequencer was processed using Illumina's RTA and CASAVA pipeline software, which includes image analysis, base calling, and sequence quality scoring, as well as pre-existing GRC-developed pipelines for sequence assessment and quality control. Following basecalling by Illumina pipelines, the quality control pipeline assessed basecall quality, converted Illumina quality scores (ASCII+64) to Sanger-style/Phred-like scores (ASCII+32) and truncated reads where the median Phred-like quality score fell below Q20. The pipeline also plotted base composition by cycle/position to identify potential cycles with aberrant chemistry errors or sample bias. These processes were parallelized using a high-performance Sun Grid Engine computer grid, which contains more than 1000 cores and five terabytes of available RAM. Further analysis of the resulting sequence reads were performed using our human exome analysis pipeline based upon the Burrows-Wheeler Aligner (BWA), and the Genome Analysis Toolkit (GATK) [21]–[23]. This process is summarized in **Figure 1**.

Initial alignment to the human reference genome (hg18) using BWA was followed by statistical analyses that calculated the percent of reads uniquely mapping, the percent of properly paired reads, and statistics that evaluate the exome capture efficiency. These included percent of mapped reads within targeted regions and within a single library-fragment-length of targeted regions (+/−200 bp). We also calculated coverage of targeted regions and percent of targeted regions that met multiple coverage criteria (5×, 20×, 30×). The resulting alignments were stored in BAM file format and we then used the Samtools package to identify and remove sequence reads that resulted from duplication of library fragments. These duplicated library fragments were artifacts of the necessary PCR step in library construction and are easily identified by having the same start and stop position in the alignment. Typical rates of duplication ranged between 10–20%. Removal of these duplicated reads prevented artificially high coverage and quality calculations and reduced false-positive variant calling.

Because Illumina quality scores tend to be over-inflated, after removal of PCR duplicate reads, the Genome Analysis Toolkit [22] performed a quality recalibration of each sequence read using locally aligned reads relative to the reference genome. The algorithm finds positions in the alignment where the sequenced base does not concur with the reference and builds an empirical probabilistic error model. Positions with known variants included in dbSNP (build 130) are ignored to minimize their contribution to basecall error calculations. Following this quality recalibration, the root mean square error (RMSE) is significantly reduced, indicating a high correlation between quality values modeled by GATK and observed quality. The quality-recalibrated data is then checked for indel positions where mis-alignments are frequent. The GATK re-aligner performs localized multiple-sequence-alignments to reduce these mis-alignments and thereby reduce errors in downstream variant detection.

Following these data quality control and pruning steps, variant detection (SNPs and indels) was performed using samtools. SNPs are called using a stringent set of quality criteria, including a minimum SNP quality score greater than 50, an allele balance less than 0.75, and a maximum of two SNPs within any 10 bp window. SNPs passing these quality criteria and falling within the targeted capture regions are stored in VCF format and annotated according to the RefSeq genes and isoforms in which they are found. The SNPs are further annotated according to classification, including coding-synonymous, missense, nonsense, intronic, UTR-5′ and UTR-3′. These classifications can be made at the isoform level to detect variants with differing potential functional impact on alternatively spliced gene products.

The analyzed exome data set, including both VCF and BAM files, can be visualized using the Broad Institute Integrated Genomics Viewer (http://www.broadinstitute.org/igv/ - accessed 2010 July), which enables display of tracks including read alignments, coverage levels, and SNP calls from multiple samples simultaneously alongside gene annotations. This provides a powerful tool for detailed comparative analysis between samples. Final data included raw sequence files, alignment files, SNP databases, and Integrated Genomics Viewer visualization.

### Exome Analyses

For clarity, and to avoid replication of descriptive text, we will sequentially describe our exome analytical methods with the associated results in the Results section.

## Results

### Source population

The GEOS and SWISS study populations served as the source population from which the 10 exome samples were selected. On the basis that the 8 of the 10 exome samples were from the GEOS study and that GEOS GWAS analyses were used to guide some of our exome analyses (to be described), additional information regarding the GEOS population is included here. The GEOS study population includes 1816 participants, consisting of 889 cases and 927 controls with the population characteristics demonstrated in **Table 1**. The mean age is 41.3 years for cases and 39.6 years for controls (P<0.001). The population is primarily composed of two self-reported race groups, European-Americans (54.5%) and African-Americans (40.4%), with the remaining 5.1% of individuals comprising other races including Chinese, Japanese, other Asians and other unspecified. There were more males than females among both cases and controls. Cases were more likely than controls to report having prevalent hypertension, diabetes and myocardial infarction and being current smokers.

The characteristics of the 10 selected cases for exome sequencing are listed in **Table 2**. While the overall goal of our 10 exome pilot was to demonstrate feasibility (i.e. capture, sequencing, bioinformatics, data management, etc.) and obtain pilot data for a potential larger project, we were also cognizant that this data provided the opportunity to develop and institute our analytic methods; albeit on a small case-only sample.

### Human Exome Capture & Illumina Sequencing Methods and Statistics

As described, we captured and sequenced ten stroke exomes. To evaluate the efficiency and success of our targeted capture and high-throughput sequencing of all human exons, we constructed paired-end Illumina sequencing libraries from whole human genomic DNA extracted from our ten clinical samples, using Agilent's SureSelect 37 Mb All Exon Kit to capture exonic DNA fragments [3], and sequenced each library using a single flowcell lane on a 75 bp paired-end Illumina run. The sequencing generated an average of 25.5 million read pairs (75 bp×2) and 3.8 Gbp per sample. We performed whole-genome alignments of the sequence reads using BWA [21], [24] against UCSC Human Genome build 18 (NCBI build 36.1) and evaluated the exome capture method using these results. An average of 97% of reads from each sample aligned uniquely and 96% of paired reads were properly mated based on orientation and distance. The sequencing and alignment statistics are demonstrated in **Table 3**.

**Table 3:** Sequencing and Alignment Statistics for Ten Stroke Exomes.

Sample ID	M006	M0114	M0379	M0432	M0823	M1012	M1096	M1107	SW393	SW708
Total Reads (75/76 bp) in millions(rounded)	43,863,768	37,155,698	40,247,747	39,894,677	34,450,161	41,907,790	39,676,183	34,271,695	40,443,932	44,516,721
Mapping %	98.18%	96.64%	98.10%	95.69%	97.41%	96.58%	96.49%	97.34%	98.30%	98.48%
Good pairs %	97.50%	96.01%	97.12%	94.90%	96.90%	95.89%	95.79%	96.92%	97.75%	98.08%
Singletons %	0.55%	0.53%	0.66%	0.74%	0.45%	0.65%	0.65%	0.39%	0.49%	0.32%
% reads in targeted regions	49.74	53.92	54.10	54.94	61.36	59.61	55.54	53.34	49.97	54.65
% reads in targeted regions +/−100 bp	64.55	66.54	65.77	61.80	69.39	67.09	61.96	60.48	60.39	65.07
% reads in targeted regions +/−200 bp	71.14	72.78	71.21	66.80	74.93	72.46	66.88	65.18	65.18	69.84
Overall coverage of target region	42.6×	38.58×	42.56×	41.45×	41.02×	39.62×	42.37×	43.01×	43.65	43.2×
% of targeted baits with >20× coverage	76.84	75.26	79.22	78.20	78.56	77.13	78.25	77.98	78.67	76.83
% of targeted baits with >30× coverage	64.37	57.81	65.88	64.07	63.60	61.15	65.06	64.98	66.37	65.29
% of baits with no reads aligning	0.76	0.56	0.44	0.62	0.70	0.71	0.67	0.72	0.49	0.95

Though the targeted capture method enables a high rate of enrichment for the targeted regions, non-specific capture also occurred. In our pilot samples, an average of 55% of aligned reads mapped to the targeted regions of the genome and 71% mapped onto or within 200 bp of the targeted regions. These rates of successful capture meet or exceed the expectations set by previous studies [3], [20]. Because of the high rates of exon capture efficiency and sequence mapping, the average depth of coverage per targeted region was 59× and less than 1% of exons went uncaptured. More than 80% of targeted exons exceed 20× coverage, which serves as a typical lower limit for high-confidence SNP calling algorithms using Illumina short-read sequence data. These high-quality and consistent data demonstrate the efficacy of the targeted capture and sequencing using the Illumina platform within the Genomics Resource Center and will serve as the basis for further refinement of the data pipeline and analysis methods.

### Data cleaning and SNP Calling Pipeline

After sequencing, our data went through several data cleaning and alignment steps in preparation for SNP calling; these procedures were explained in the Methods. The application of these methods to our 10 exomes resulted in SNP calling as denoted in **Table 4**. After applying quality filters, African-Americans showed greater variation with an average of ∼24.5 K variants compared to ∼22.4 K for Caucasians as expected from the literature; the number of variants (not in dbSNP) was 2839 (11.6%) vs.1105 (4.9%), respectively. Although we have predicted the indels, we have not yet analyzed them.

**Table 4:** SNP Summary for SNPs in target exome regions only.

SAMPLE ID	M006	M0114	M0379	M0432	M0823	M1012	M1096	M1107	SW393	SW708
**SNP's**	31,990	31,097	31,756	31,255	32,368	31,385	30,942	31,595	26,472	26,942
**#SNPs in dbSNP**	27,364	26,887	27,506	26,260	27,139	26,198	26,045	26,797	24,116	24,509
**Pass Q Filters**	28,103	26,844	28,205	26,668	28,022	26,787	26,449	27,574	23,262	23,697
**Pass QF and in dbSNP**	25,097	24,486	25,370	23,899	24,935	23,830	23,674	24,651	22,182	22,567
**Pass QF and Novel**	3,006	2,358	2,835	2,769	3,087	2,957	2,775	2,923	1,080	1,130
**Indels**	8,313	6,033	6,583	4,750	4,909	5,193	4,737	4,823	5,214	5,599

**Figure 2:**
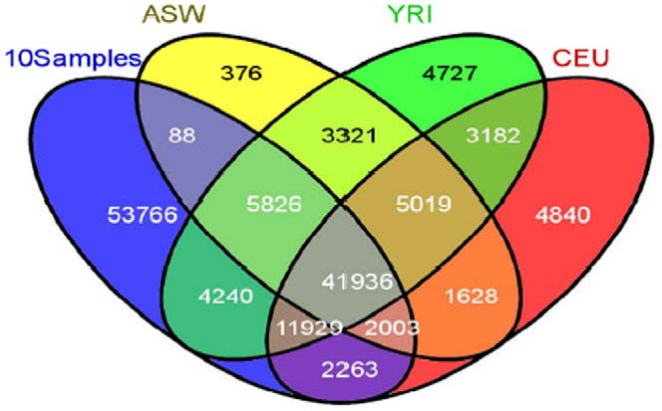
SNPs in the 10 cases exomes vs. 3 control populations.

To further evaluate the quality our data we sought publically-available exome (genome) data that could be used for comparison purposes. Here it is important to note that while one might consider utilizing only publically-available unaffected control reference data for comparison with our case-only exome data, such methods may introduce unbeknownst errors. While such methods may be appropriate when studying rare Mendelian diseases that express a highly-defined phenotype, complex diseases by nature have a less well-defined phenotype, with the genetic contribution to any specific complex disease being un-established across any potential publically-available control population. As such we propose that unaffected matched controls to the cases under evaluation be utilized along with the publically available control data, then statistically evaluating for differences in the controls population prior to case-control comparisons. In our pilot study no matched controls were utilized, however 3 appropriate publically-available control populations were identified in dbSNP. These included 61 African Ancestry individuals from the Southwest US (ASW), 100 Yoruba (Ibadan, Nigeria (YRI)) and 116 Caucasians of Western European ancestry (CEU), all of which were sequenced as part of the 1000 Genomes Project (http://www.1000genomes.org/about - accessed 2011 July). Comparisons of ***all*** SNPs identified in our 10 exome study subjects and those within the same exomic regions from subjects within the 3 controls populations are seen in **Figure 2** and denoted in the first line of **Table 5**. In summary, there is an overlap of 41936 SNPs among the 4 populations (of note these are not unique SNPs, but are SNPs that appear at least once in any individual in the population). Our cumulative exome data (all 10 subjects) demonstrates 53766 SNPs that were not seen in any of the control populations (see **Figure 2**). Many of these SNPs are shared across our case samples. Comparing our exome data with these pre-existing reference populations highlights several key points. *First*, our data has significant overlap with these datasets, highlighting the quality of our exome data. *Second*, the overlap between our data and the various populations is incomplete, demonstrating that there are numerous previously unidentified variants in our exomic dataset. *Third*, the two differing African-American control populations have incomplete overlap, emphasizing the need to use well-matched controls (to the cases); implementing principal component and ancestry informative marker analyses will minimize confounding secondary to population admixture.

**Table 5:** Select Gene Analyses: 10 Exome Cases vs. 3 Control Populations.

	Exon bases	10 exome snps	10 exome rs	10 exome rs (%)	10 exome novel	ASW snps	YRI snps	CEU snps	Shared ASW-YRI	Shared ASW-10exome	Shared YRI-10exome	Shared ASW-YRI-10exome	Shared CEU-ASW	Shared CEU-YRI	Shared CEU-10exome	Shared All	Non-Common SNP density
**SNP Count Comparison**		162,201	122,052	75.2	40,149	60,197	80,180	72,800	56,102	50,586	62,066	49,853	63,931	58,131	47,762	41,936	
**Lacunar Genes as selected per GWAS**																	
***CSN3***	1312	**10**	7	70.0	3	4	5	5	4	3	4	3	4	5	4	**3**	**5.3**
*FLT3*	8622	35	28	80.0	7	10	17	17	10	10	16	10	10	16	15	10	2.9
*FMO2*	3512	20	20	100	0	19	18	12	18	16	15	15	12	12	11	11	2.7
*HLA-DPB1*	2025	46	46	100	0	6	16	13	6	5	14	5	6	13	11	5	20.3
*LRIT2*	2300	11	10	90.9	1	3	7	5	3	3	7	3	2	5	5	2	3.9
*NAT10*	9798	25	15	60.0	10	2	10	7	2	2	9	2	2	4	3	2	2.4
*PIKFYVE*	4582	8	4	50.0	4	3	3	2	3	3	3	3	2	2	2	2	1.3
*PODN*	4332	17	14	82.4	3	8	13	15	8	5	9	5	8	13	9	5	2.8
*TEKT2*	2821	7	3	42.9	4	2	3	2	2	2	2	2	1	1	1	1	2.1
**Candidate Genes**																	
*ACE*	9915	31	28	90.3	3	10	14	13	9	9	12	9	8	11	11	8	2.3
*APOE*	1782	2	2	100	0	2	2	1	1	0	1	0	0	0	0	0	1.1
*F2*	4976	8	7	87.5	1	8	8	9	6	4	4	3	7	6	4	3	1.0
*F5*	13243	48	42	87.5	6	34	36	44	26	22	28	21	31	28	27	20	2.1
*ITGA2*	11020	41	37	90.2	4	28	39	34	27	25	33	25	26	33	30	23	1.6
*ITGB3*	6226	21	17	81.0	4	10	12	12	9	8	9	8	10	11	9	8	2.1
*LTA*	1189	5	5	100	0	3	3	4	2	3	3	2	3	3	4	2	2.5
*MTHFR*	4666	19	18	94.7	1	10	12	13	10	10	12	10	7	9	10	7	2.6
*NINJ2*	1277	2	2	100	0	3	2	3	2	2	2	2	3	2	2	2	0.0
*NOS3*	8896	22	22	100	0	9	12	11	7	6	7	5	7	7	6	3	2.1
*SERPINE1*	3054	8	6	75.0	2	5	7	7	5	3	3	3	3	4	4	3	1.6

As a final evaluation of the quality of our exome data we compared our exome genotype calls with the available fixed GWAS content [18] genotype calls of the 8 GEOS study subjects. These results are detailed in **Table S1**. In summary, there were 29600 exonic SNPs in the GWAS data; call comparisons demonstrated a concordance rate of ∼99% across the 8 subjects.

### Analyses of the 10 Stroke Exomes

We spent considerable time and effort optimizing our SNP calling algorithms pipeline (see Methods), with the results of several different analyses utilizing our 10 pilot case exomes now presented.

### Gene–Specific Analyses

Pursuant to our hypothesis that excess rare variation may play an important role in gene-specific stroke risk, we decided to evaluate specific genes among our exome data. Before we could embark on such analyses, control data was required and was obtained as described above. Two sets of genes were chosen for these analyses. The *first* were the genes containing the “top" lacunar stroke coding variants as identified by our GWAS study and the *second* were a series of candidate genes selected from the literature [25]–[27].

The ‘top’ lacunar stroke coding variants from our GWAS results were identified by sorting our lacunar stroke specific results (lacunar stroke cases vs. all controls) by p-value smallest to largest. This was performed in the pooled sample and by ethnicity (African-Americans and Caucasians). Our hypothesis was that while none of these common variants reached genome-wide significance, maybe these gene-specific variants were marking stroke susceptibility genes and that potentially unknown excess rare-variation could also play a role. To be clear, we do *not* believe that common variation is marking rare variation, but hypothesize that potentially a combination of common and rare variation in these genes may work together to increase risk. Utilizing our GWAS results [18] for lacunar stroke subtype we screened for the 1000 smallest p-values from each of the 3 groups (pooled sample and by ethnicity (African-Americans and Caucasians)), then further screening each list to identify the *nonsynonymous* coding SNPs present. This was performed implementing SIFT [28] (http://sift.jcvi.org/www/SIFT_dbSNP.html - accessed 2010 July) which identified the *nonsynonymous* coding SNPs (missense only; no stop codons were seen) and also rated the variants as deleterious or not. Among the pooled, African-American and Caucasian groups respectively, 17, 14 and 17 missense variants were identified; of these 4, 3, and 2 were considered to be deleterious, inducing amino acid changes purported to affect protein function. We then identified the genes containing these variants. Of note, there were no SNP (or gene) overlaps between the two ethnicities. **Table 5** shows the identified genes.

The candidate genes selected for our second set of analyses were chosen on the basis that they harbor common genetic variants associated with ischemic stroke that have positively replicated in follow-up studies [25]–[27] or that underwent additional analyses as part of our GWAS [18]. These genes are listed in the lower portion of **Table 5** and include: ***MTHFR***, ***F5***, ***F2***, ***ITGB3***, ***ITGA2***, ***LTA***, ***SERPINE1***, ***ACE***, ***ApoE***, ***NOS3***, and ***NINJ2***.

Gene-based analysis of our 10-case exome data for both sets of genes (‘top’ GWAS lacunar hits and Candidate) as compared to the control populations were then initiated. Of note, we felt a combined analysis (8 African-American and 2 Caucasian) was appropriate given our limited number of exomes and pursuant to the hypothesis that shared risk mechanisms may exist across ethnicities. First, we screened the exome data of our cases to identify both the known (common and rare) and novel (rare) variants in the coding regions of each gene, followed by a screening comparing variant counts and overlap between our data and the control populations as shown in **Table 5**. Assuming that excess variation in a gene may play a role in risk, from these data we calculated the non-common-SNP density per Kb by gene (i.e. (total exome variants - shared variants across all 4 populations)/exon base-pairs) of which *HLA-DPB1* and *CSN3* (e.g. 10-3 = 7; 7/1.312 Kb = 5.34/Kb) had the greatest values. Given the expected high variability of HLA genes, only *CSN3* (Kappa-Casein) SNPs were further evaluated. Among the ten *CSN3* SNPs in the exome data, three were previously unidentified as per dbSNP, each with an allele count (AC) of 5% (i.e. 1 in 20). Notably, two of these three SNPs were classified as non-synonymous annotated using the VcfCodingSnps tool developed at University of Michigan (http://genome.sph.umich.edu/wiki/VcfCodingSnps - accessed 2010 July). Of the remaining seven established SNPs (i.e. in dbSNP), three were not seen in any of our control groups; corresponding ACs of 15%, 5%, and 5%. Of the remaining four SNPs seen in both the exome and control data, two are also non-synonymous SNPs both with AC = 5%; one is intronic with AC = 5%; the last is synonymous with AC = 20%. Overall, these allele counts are similar to the minor allele frequency as seen in the Control groups if present. We are currently having several International Stroke Genetics Consortium (ISGC) collaborators (http://www.strokegenetics.org/ - accessed 2011 July) evaluate their GWAS data with respect to this gene.

### Compound Heterozygote Analyses

Pursuing the hypothesis that stroke susceptibility genes may be enriched for novel variants across cases, we screened the exome data for genes in which every case had a least one rare variant. This methodology identified 70 gene isoforms where each African-American case had at least one rare variant, with this number dropping to 48 when the 2 Caucasian cases were also included. Taking this a step further by implementing a “double-hit hypothesis" (i.e. compound heterozygote) to evaluate for two novel variants in the same gene isoform, only 6 genes (9 total isoforms) satisfied this criterion. Three of the six genes identified are involved in various forms of metabolism (*AQP7*, *CTBP2*, *ARSD*), and of these, one was previously associated with diabetes (*AQP7*). Of the three remaining genes, one is involved in the inflammatory response (*HLA-A*), one is solely expressed in the CNS (*OR4C3*), and the function of the last is unknown (*FRG2C*). *CTBP2* had three isoforms identified while *ARDS* had two. Counting the number of novel mutations (2 or more) per each gene isoform per sample is shown in **Table 6**.

**Table 6:** Genes in which at least two variations which are novel.

	Sample ID									
Gene/Isoform	M006	M0114	M0379	M0432	M0823	M1012	M1096	M1107	SW393	SW708
OR4C3/NM_001004702	6	4	4	4	3	5	4	10	4	5
CTBP2/NM_001083914	8	7	5	10	7	8	9	7	7	9
FRG2C/NM_001124759	7	6	8	7	5	8	6	8	11	7
AQP7/NM_001170	6	8	11	8	7	6	8	4	6	7
CTBP2/NM_001329	8	7	5	10	7	8	9	7	7	9
ASRD/NM_001669	10	9	9	9	9	10	7	11	11	11
HLA-A/NM_002116	6	5	7	7	8	6	2	4	3	5
ASRD/NM_009589	9	9	9	9	9	10	7	11	11	11
CTBP2/NM_022802	6	6	3	7	5	7	7	6	5	8

**Figure 3:**
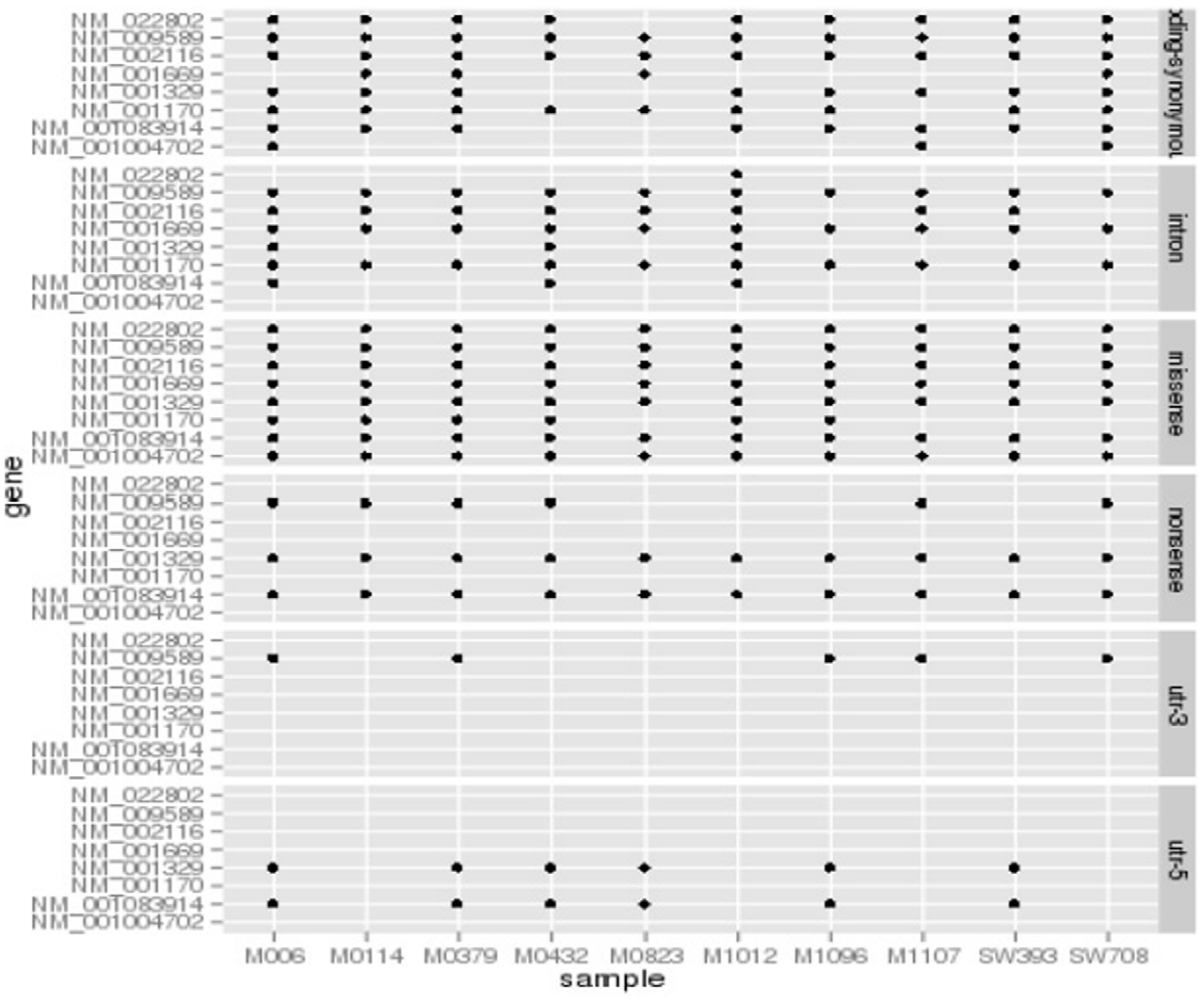
Compound heterozygotes in exome data for genes in which every case had a least two novel variants in the same gene isoform, only 6 genes (9 total isoforms) satisfied this criterion. The figure illustrates the 9 isoforms (left y-axis) per sample (x-axis) by variant type (left y-axis). Notably, two of the *CTPB2* isoforms (NM_001329 and NM_ 001083914) were seen to have a non-sense codon occurring in all 10 samples.

Our first impression of these numbers was that they seemed high. Nevertheless, we continued our analyses to further classify these variants by type as demonstrated in **Figure 3**. In this figure the variants per sample (x-axis) are classified by type (right y- axis) and per gene isoforms (left y-axis). Notably, two of the *CTPB2* isoforms (NM_001329 and NM_ 001083914) were seen to have a non-sense codon occurring in all 10 samples.

**Figure 4:**
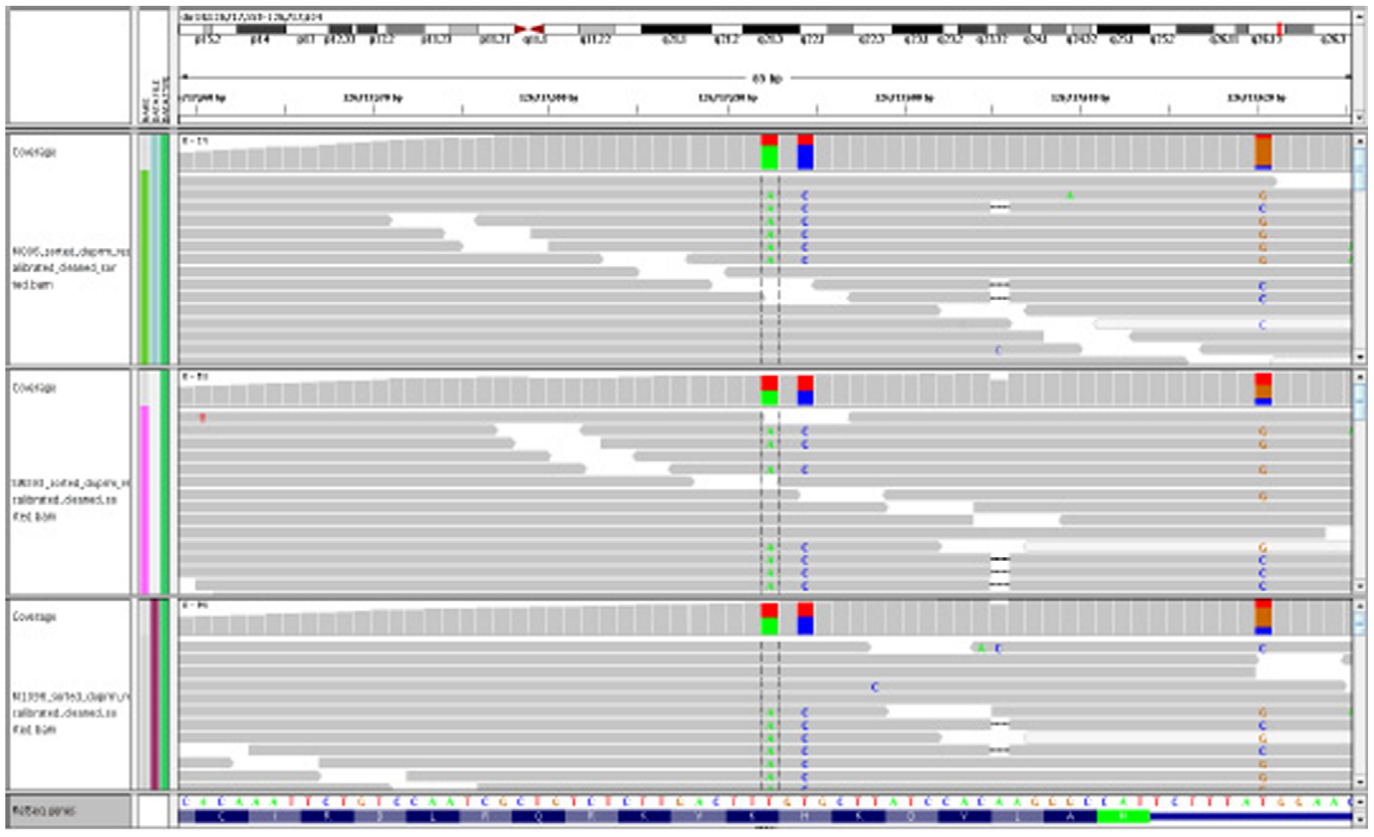
This image shows the start region for *CTPB2* gene (chromosome 10) in the Integrated Genomics Viewer. The top three tracks show aligned sequence coverage for three of the pilot exomes and demonstrate the T to A substitution resulting in the nonsense codon as compared to the two bottom tracks showing the annotated RefSeq and codon positions.

Exploring these two *CTPB2* isoforms, the same variant in all 10 samples was identified occurring at basepair position 126717592 resulting in T to A substitution [TTT, TTA] and a LYS to nonsense codon in position 8 of the protein. This *CTBP2* region is graphically demonstrated in **Figure 4** (using the Broad Institutes' Integrated Genomics Viewer (IGV) (http://www.broadinstitute.org/igv/ - accessed 2010 July), which displays the read alignments, coverage levels, and SNP calls from multiple samples simultaneously (3 in this case) alongside gene annotations. Pertinent to our *CTBP2* data, we see that in the three sample tracks shown the aligned sequence coverage demonstrates the T to A substitution resulting in the nonsense codon as compared to the two bottom tracks showing the annotated RefSeq and codon position. While finding a novel stop codon among our cases would be a highly important finding, we believed that this finding is due to non-specific capture of a non-exome region that has a similar sequence to our exome region of interest, in other words this is a gene-duplication artifact. In explanation, all the cases were heterozygote at this location. Further, the A–C haplotype as seen in **Figure 4** indicates two novel SNPs (codon 7 and 8), however upon screening dbSNP both of these SNPs have been identified in the whole genome sequence of a Korean and in dbSNP (http://www.ncbi.nlm.nih.gov/projects/SNP/snp_ref.cgi?chooseRs=coding&go=Go&locusId=1488; following links on the rs numbers one finds that “Lee" submitted both rs76555439 and rs78849795 - accessed 2011 January). Further evaluating **Figure 4** one can see that there is a SNP with 3 base variations at the far right, this finding would not be impossible, but would be unusual. Note that the indel is perfectly correlated with the C allele. Lastly, Mendelian diseases tend to be highly heterogeneous in causal alleles, as seen in the recent exome success stories. Finding the same causal allele for a complex disease across both ethnicities and among the various stroke subtypes evaluated would be very unusual. As such, here we mention a few observations and potential conclusions regarding this specific finding. First, we detected a duplication in the genome that is either not annotated or picked up by the current alignment/unique mapping filter. This duplication is probably monomorphic A/A and C/C at the first two sites, but appears to have perfectly correlated SNPs at the indel and C/G site. This nonsense mutation would not have shown up as novel if filtered by current dbSNP, but the mutation would have been picked up filtering by genes with nonsense mutations in (multiple) cases. When filtering missense/nonsense SNPs by dbSNP it might be useful to be able to filter by allele frequency; If ND (not determined) then potentially keep. As dbSNP is more regularly updated with exome and whole genome data, then filtering will require care as a stroke variant may be present in a subject from a non-stroke study.

## Discussion

Our exome capture, sequencing, and analyses are among the first to examine for the presence and affect of rare variation in ischemic stroke. Given that the optimal methodologies regarding exome analyses are not fully-established in the study of complex disease, including stroke, we developed a variety of analyses to examine our exome data. First, pursuant to our hypothesis that excess rare variation may play an important role in gene-specific stroke risk, we evaluated specific genes chosen based upon our predominant stroke subtype, lacunar stroke, and also by selecting genes based upon prior candidate gene studies. The exomic analyses informed upon by our lacunar stroke GWAS results identified two genes, *CSN3* and *HLA-DPB1*, which appeared to have excess variation in our stroke cases. Further analyses of *CSN3* are ongoing; however given the expected high variability of HLA genes, no further analyses of *HLA-DPB1* has occurred. None of the previously identified candidate genes studied demonstrated ‘excess’ rare variation in our exome samples. A compound heterozygote analyses pursuant to the hypothesis that stroke susceptibility genes may be enriched for novel variants across cases was also performed, identifying several genes with rare variation across all 10 cases. In particular, *CTPB2* appeared to contain a nonsense codon that appeared in all 10 cases, however after further analyses this was identified as a false positive. As a pilot study we identified several important methodological considerations and pitfalls that should be considered when implementing such methods. First, we emphasize the importance of selecting a highly defined phenotype among the study subjects, such as selecting on one stroke subtype, few established vascular risk factors, and positive a family history of stroke. Further selecting the study subjects in the extremes of the phenotypic tail (i.e. young-onset stroke) may also further maximize the genetic contribution to stroke risk. Second, as our results demonstrate, investigators must evaluate for the presence of potential false positive findings, a major pitfall regarding this type of research. Utilizing existing databases for non-diseased control samples may be fraught with difficulties as a control populations ascertained to study a different disease may contain an individual with the disease under study (in this case stroke). When studying rare variation, introducing such an error would be particularly troublesome.

While our gene specific analyses failed to identify excess rare variation in any of the ischemic stroke candidate genes evaluated, our analyses evaluating our exome data in consideration of our prior GWAS results identified one gene, kappa-casein (*CSN3*), which warrants additional discussion. As described, by screening our prior GWAS results for genes containing coding SNPs in lacunar stroke, and then evaluating the implicated genes in our exome data, we identified *CSN3* (OMIM: 601695; http://omim.org/entry/601695 - accessed 2011 July) as containing an interesting coding polymorphism as well as containing excess rare variation. *CSN3* maps to chromosome 4 at 4q21.1 and contains 5 exons covering 8.85 kb on the direct strand from position 71108305 to 71117153 (NCBI 37, August 2010). The predicted protein has 182 aa (20.3 kDa, pI 8.1). While the function and tissue expression pattern of this gene is are not fully established, Kappa-casein is known to stabilize micelle formation preventing casein precipitation in human breast milk (http://www.genecards.org/ - accessed 2011 July). Expression studies have demonstrated the presence of kappa-casein in a variety of soft tissue/muscle tissue cancers [29] and also in coronary artery atherosclerosis [30]. In the coronary atherosclerosis study, the investigators performed a comprehensive gene level expression assessment of coronary atherosclerosis using 51 coronary artery segments isolated from the explanted hearts of 22 cardiac transplant patients, demonstrating that *CSN3* was consistently highly expressed in all arterial segments analyzed.

Other studies have indicated that kappa-casein may play a role in immune and inflammatory responses via regulation of the transcription factor nuclear factor kappaB (NF-kappaB) [31]. Activation of NF-kappaB requires the activity of IKK, a kinase complex that contains two catalytic subunits, IKKalpha and IKKbeta, and a regulatory subunit IKKgamma. In one such study, the investigators worked to understand how IKK activity was regulated, and searched for IKKgamma-interacting proteins utilizing a ‘yeast two-hybrid system’. Screening identified CSN3, a component of the COP9 signalsome, as a protein specifically interacting with IKKgamma. Over-expression of *CSN3* inhibited NF-kappaB activation triggered by tumor necrosis factor (TNF), but not interleukin-1 (IL-1). Moreover, over-expression of *CSN3* also inhibited NF-kappaB activation triggered by proteins involved in TNF signaling, including TNF-R1, TRAF2, RIP, and NIK, but not by TRAF6, a protein involved in IL-1 signaling. These data suggest that CSN3 is a specific negative regulator of TNF- but not IL-1-induced NF-kappaB activation pathways.

*CSN3* was also identified as a novel candidate genes for type 2-diabetes mellitus (T2-DM) in a genome-wide association scan in the Old Order Amish [32]. In this study of 124 type 2 diabetic case subjects as compared with 295 control subjects, *CSN3* SNP rs3775745 was found to be associated with T2-DM in the Amish (p = 0.002), with this result replicating in a Mexican-American population (p = 0.003). Of note, none of our exome study subjects had diabetes.

Exome based approaches offer different, yet complimentary, information to ongoing stroke genome wide association study (GWAS) efforts, including ongoing projects implementing substantially larger samples sizes as compared to our GWAS sample. While we are optimistic about the success of GWAS to identify stroke associated variants, we do not believe that GWAS results will account for all stroke risk. This point of view was recently supported by the results of a Cohorts for Heart and Aging Research in Genomic Epidemiology (CHARGE) Consortium GWAS study evaluating ischemic stroke risk in which only 2 intergeneic SNPs were found to achieve genome-wide significance [27]. In the CHARGE study, SNP rs12425791 on chromosome 12p13 was associated with an increased risk of ischemic stroke with a hazard ratio of 1.33 (95% CI, 1.21 to 1.47), yielding a population attributable risk of 12%. A corresponding hazard ratio of 1.42 (95% CI, 1.06 to 1.91; p = 0.02) was attained in a large follow-up cohort of black subjects. While these results were extremely important, they did not demonstrate that a few common variants account for the majority of stroke risk. Furthermore, our research group and several other groups participating in the ISGC failed to replicate the findings of the CHARGE study, thereby calling into question the validity of the CHARGE results [33].

While genome-wide association studies (GWAS) can identify common variants, they are not suited for situations where genetic architecture is such that multiple rare disease-causing variants contribute significantly to disease risk. This is because GWAS chips most often implement common variants as identified through the HapMap project and these variants do not serve as markers for rare variation [34]. As such, we believe that sequencing will ultimately identify rare risk variants and that exome sequencing, given its ability to identify rare variants of high penetrance, is an excellent methodology to begin these efforts. Large scale exome projects are now well underway, most notably including the National Heart, Lung, and Blood Institute (NHLBI) GO Exome Sequencing Project (ESP). The ESP is funded by NHLBI and managed by both NHLBI and National Human Genome Research Institute (NHGRI). The goal of the ESP is to develop and validate a cost-effective, high-throughput sequencing application for all protein coding regions of the human genome in the study of several diseases, including ischemic stroke. The purpose of developing this resequencing application is to enable the sequencing of tens of thousands of individual samples from NHLBI's well-phenotyped populations in a cost-effective manner. These data will be publically-available and free to use by the scientific community. Among these there will be a deeply phenotyped reference sample not selected on basis of disease, consisting of 750 Caucasians of European origin and 250 African-Americans. The first samples have recently been submitted to dbGaP with many more over the next year.

Our pilot study has several limitations. Most notably the small sample size which was directly attributable to the costs associated with exome sequencing. Further, given that stroke is a prototypical complex disease with numerous risk factors and subtypes, the genetic mechanisms of each stroke (by subtype) included in our study is likely somewhat different. However, as described in the Methods section, we worked to limit the presence of risk factors in our young-stroke onset samples. Our study was also hampered by a lack of standardized analysis programs for exome analysis. At present the analytic methodologies regarding rare-variant analyses are an area of active methodological research [35]. While there were certainly many different potential analyses that could be performed on our dataset, we focused our efforts on what we felt might yield the ‘low lying fruit’; that is, non-synonymous coding variants leading to missense or nonsense mutations. This was motivated by the fact that over half of all known disease mutations come from such replacement polymorphisms; such an assumption may or may not be appropriate in the setting of ischemic stroke. Here we also highlight that our definition of “non-common variants" refers to variants that are not shared between the exome data and the combined 3 control populations. For example, a shared variant between the African-American control data (YRI or ASW) and the 8 African-American exome cases, that was not seen in the Caucasian control data, would be considered “non-common". The reasoning for formulating our definition in this fashion was that we were seeking a shared gene-specific stroke mechanism across ethnicities rather than an ethnicity specific mechanism. In other words, we were seeking global excess variation among the cases in the genes evaluated as compared to control population as a whole. While it would certainly be interesting to perform an ethnicity stratified analyses, we felt that our current sample size precludes such analyses. Another important analysis issue we considered is the potentially confounding effect of population substructure which could induce false-positive (or false negative) findings. Implementing PLINK [36], [37] on our GWAS data allowed us to identify 47 population outliers who were removed from the pool considered for our exome analyses. Lastly, by including African-Americans in our exome analyses we inherently complicated our study. While there is little data available on the allelic spectrum of the exome in African-Americans, HapMap data has shown up to a 2 fold greater number of SNPs in the Yoruba (Ibadan, Nigeria) (YRI) same set samples compared to the Caucasians (CEU). However, given the fact that African-American adults are twice as likely to have a stroke as their Caucasian adult counterparts [38], with this especially true in the young [39], [40], we felt that the additional scientific opportunity offset the additional exome complexity inherent when studying African-Americans.

Our study also has several advantages. First, our study population is an ideal sample to evaluate since young age-of-onset is an extreme phenotype that is likely enriched with rare variants; further supported by the fact that numerous familial aggregation studies implicate a greater genetic risk at younger ages. Another strength is that our exome samples were also genotyped on the Illumina 1 M Quad [Omni Quad] as part of our groups' participation in the GENEVA consortium [41], thereby allowing us to perform the combined exome and GWAS analyses as described. Of note, our GWAS data was rigorously evaluated by the GENEVA Data Cleaning Group (University of Washington, Seattle, WA) and the genotyping center, the Center for Inherited Disease Research (CIDR) (http://www.cidr.jhmi.edu/ - accessed 2011 July). Quality metrics include missing genotype call rates by sample and SNP, Hardy-Weinberg analysis, and detailed global and local population substructure analysis using principle components analysis [18].

In closing, our study evaluated a highly prevalent complex disease for which the genetic architecture remains uncertain. Prior and ongoing studies implementing genome-wide association techniques are only capable of identifying common variants associated with disease. The extent to which ischemic stroke is determined by rare or low frequency variants has not yet been explored. Our study demonstrates the feasibility of utilizing exome based techniques to address this important research gap by implementing several different analyses at the variant and gene levels. Our study also highlights several of the important considerations in this type of research, such as attaining a highly specific phenotype and utilizing an extreme phenotype that is likely enriched with rare variants; in our case young-onset subtype-specific stroke. One gene, *CSN3*, identified by screening our prior GWAS results for genes containing coding SNPs in lacunar stroke, was found to both contain an interesting coding polymorphism as well as excess rare variation in our exome data as compared with the other genes evaluated. Additional research will be required to determine if *CSN3* variants are truly associated with ischemic stroke risk. Lastly, while rare coding variants may predispose to the risk of ischemic stroke, this fact has yet to be definitively proven. Our study demonstrates the complexities of such research and highlights that while exome data can be obtained, the optimal analytical methods have yet to be determined.

## Supporting Information

Table S1 Call rate concordance between overlapping exome and GWAS SNPs.
(DOC)
