# Supplementary material for: Rare Variants in Ischemic Stroke: An Exome Pilot Study
Source: PLoS One. 2012 Apr 20;7(4):e35591. doi: 10.1371/journal.pone.0035591 (PMC3334983; doi:10.1371/journal.pone.0035591)
Supplement: Table S1 — Call rate concordance between overlapping exome and GWAS SNPs. (DOC) [file pone.0035591.s001.doc]

**Supplementary Table 1.** Call rate concordance between overlapping exome and GWAS SNPs.

| **Sample** | **Agree** | **Disagree** | **Sequencing**  **NoCall** | **Disagree**  **excluding**  **NoCall** | **Total**  **Overlap** | **Concordance** |
| --- | --- | --- | --- | --- | --- | --- |
| **M0006** | 29232 | 368 | 110 | 258 | 29600 | 99.13% |
| **M0114** | 29292 | 308 | 67 | 241 | 29600 | 99.18% |
| **M0379** | 29320 | 280 | 68 | 212 | 29600 | 99.28% |
| **M0432** | 29127 | 473 | 161 | 312 | 29600 | 98.94% |
| **M0823** | 29226 | 374 | 108 | 266 | 29600 | 99.10% |
| **M1012** | 29073 | 527 | 195 | 332 | 29600 | 98.87% |
| **M1096** | 29098 | 502 | 162 | 340 | 29600 | 98.85% |
| **M1107** | 29225 | 375 | 133 | 242 | 29600 | 99.18% |
| **Average** | **29,199** | **401** | **126** | **275** | **29,600** | **99.07%** |
